# Extraordinary *Trypanosoma cruzi* diversity within single mammalian reservoir hosts implies a mechanism of diversifying selection

**DOI:** 10.1016/j.ijpara.2010.12.004

**Published:** 2011-05

**Authors:** Martin S. Llewellyn, John B. Rivett-Carnac, Sinead Fitzpatrick, Michael D. Lewis, Matthew Yeo, Michael W. Gaunt, Michael A. Miles

**Affiliations:** a The London School of Hygiene and Tropical Medicine, Keppel Street, London WC1e 7HT, UK; b Lion House, 23 Onslow Road, Richmond, Surrey TW10 6QH, UK

**Keywords:** *Trypansoma cruzi*, Chagas disease, Microsatellite, Infrapopulation, Multiclonality, Didelphis

## Abstract

*Trypanosoma cruzi* is an evolutionarily ancient parasitic protozoan endemic to the Americas. Multiple genetic and phenotypic markers indicate that this parasite is highly diverse, with several divergent and discrete major genotypes reported. Infection multiclonality has been observed among numerous metazoan and unicellular endoparasitic species. However, few studies report the complexity of mixed infections within an individual host in any detail or consider their ecological and biological implications. Here we report extraordinary genetic diversity within single reservoir hosts of *T. cruzi* I using nine polymorphic microsatellite markers across 211 clones from eight mammals from three different sylvatic foci in South America. Forty-nine distinct multilocus genotypes were defined, with as many as 10 isolated from the same host. We discuss our data in the light of previous population genetic studies of this and related parasitic protozoa and contrast high levels of diversity within each host with the precarious nature of *T. cruzi* contaminative vectorial transmission. Finally, we propose that non-neutral processes could easily account for the diversity we observe and suggest a functional link with survival in the host.

## Graphical abstract

**Figure:**
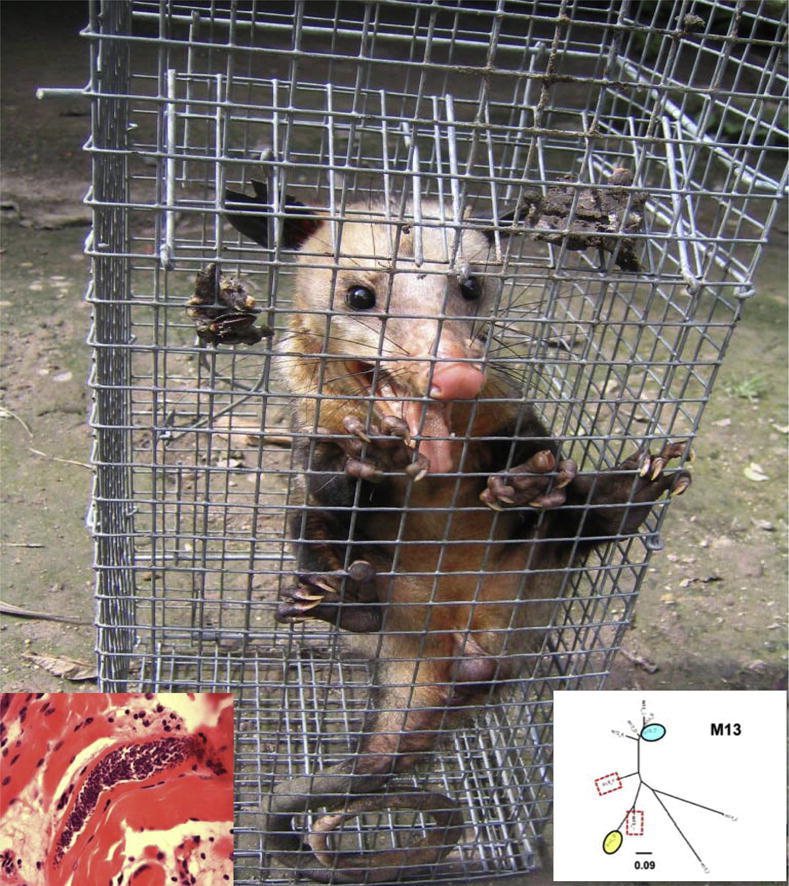

**Research highlights:**

► We report extraordinary genetic diversity within single reservoir hosts of Trypanosoma cruzi. ► Two hundred and thirty-three biological clones were taken from eight mammals. ► Nine polymorphic microsatellite markers were amplified. ► Forty-nine distinct multilocus genotypes were defined. ► Widespread multiclonality contrasts with the precarious nature of T. cruzi vectorial transmission. ► We propose that non-neutral processes could account for the diversity observed.

## 1 Introduction

*Trypanosoma cruzi*, the etiological agent of Chagas disease, is ancient and endemic in the Americas. The progenitors of this digenic kinetoplastid are thought to date their origins to the breakup of Gondwana and were, therefore, probably parasites of South America’s earliest mammalian fauna (Stevens et al., 1999). As such, *T. cruzi* is common in sylvatic cycles of transmission throughout the continent where numerous species of native mammal and haematophagous triatomine vector maintain the parasite.

*Trypanosoma cruzi* is highly genetically diverse. Six major genotypes or ‘Discrete Typing Units’ (DTUs) are recognised to date, newly dubbed TcI–TcVI (Zingales et al., 2009). TcI, TcIII and TcIV are by far the most common in wild transmission cycles (Yeo et al., 2005; Miles et al., 2009). Among domestic transmission cycles, TcI predominates in northern South America, where it may be associated with less severe human disease, while TcII, TcV and TcVI are more common in the Southern Cone, where Chagasic megasyndromes are more frequent (Miles et al., 2009). Among TcI and TcIII isolates at least, genetic diversity at the sub-species level in *T. cruzi* can be extrapolated to considerable diversity at the sub-lineage level based on microsatellite and sequence markers (Herrera et al., 2007; Spotorno et al., 2008; Llewellyn et al., 2009b; Miles et al., 2009). TcV and TcVI lineages, on the other hand, show minimal diversity across multiple markers and a large geographic area, consistent with a recent hybrid origin and rapid expansion into domestic transmission cycles (Lewis et al., unpublished data). TcII, sparsely isolated, is poorly defined in both genetic and epidemiological terms (Lewis et al., unpublished data), however, a growing collection of isolates from sylvatic cycles across Brazil looks set to change this (Lima et al., personal communication). Among TcI isolated from lowland Bolivia, Venezuela and Brazil, Poisson-distributed allele frequencies point to large, stable effective population sizes (Llewellyn et al., 2009b)

The occurrence of multiclonal infections in a single host is an almost universal expectation given measurable levels of genetic diversity in a species of endoparasite. In *T. cruzi*, hosts and vectors have occasionally been identified with mixed infections of different DTUs (e.g. (Bosseno et al., 1996; Herrera et al., 2005; Yeo et al., 2007; Burgos et al., 2008)). Preliminary data demonstrate that multiple variants of the same DTU could also be present (Macedo et al., 2001; Yeo et al., 2007). Sources of intra-host diversity include superinfection from discrete sources as well as the simultaneous transmission of multiclonal parasite populations. In both cases, clonal multiplicity of infection is likely to be related to both the intensity and efficiency of transmission, as is the case with other vector-borne protozoa, most notably *Plasmodium falciparum* (e.g. Schoepflin et al., 2009). However, genetic exchange between individuals within the host or vector is another potential source of multiclonality. One would expect de novo mutation to account only minimally for intra-host genetic diversity, given an appreciable rate of horizontal transmission. On the other hand, genetic exchange will act rapidly to generate multiple non-identical genotypes (Halkett et al., 2005), even if the overall level of genetic diversity remains low. A recent review of the evidence for genetic exchange in *Leishmania* described the diversity of clones within an individual host or vector as the possible location of the ‘demographic unit’ in a parasite population (Rougeron et al., 2010). This implies that individual clones in close physical proximity are those that are most likely to exchange genetic material.

A group of parasites of the same species inhabiting a given host has also been termed an ‘infrapopulation’ (Bush et al., 1997). Infrapopulations, including all different lifecycle stages of the parasite, can be grouped to form the ‘component’ population (Bush et al., 1997) – the level at which most micro-parasite population genetic studies operate. However, instead of examining different clones from each host separately, difficulties associated with live parasite isolation and large scale biological cloning means that the ‘individual’ in such studies is often simply DNA extracted from host tissue or primary parasite culture. This extract, therefore, represents a composite of a subset of the clones present in each vector or host (e.g. Nebavi et al., 2006; Koffi et al., 2009; Llewellyn et al., 2009a,b; Morrison et al., 2009; Rougeron et al., 2009). The theoretical consequences of sampling a single clone per host have recently been discussed and novel study designs proposed to improve the estimation of key population statistics and processes (Prugnolle and De Meeus, 2010). Lamentably, such designs are not always possible in the field as sample sizes may be inflated beyond the scope of (and available funding for) the study. The theoretical consequences of analysing the clonal composites, a more commonly encountered problem, warrants further exploration.

Here we present multilocus microsatellite data for 211 biological clones taken from eight TcI reservoir host strains. We demonstrate substantial multilocus genotype (MLG) diversity within these strains, even where multiple (⩾3) alleles at individual loci are not present in the uncloned profile and despite multiple repassages, long-term cryopreservation and different isolation techniques. We compare the resultant MLGs with those from the original, uncloned microsatellite profile with the aim of qualitatively assessing bias associated with using such profiles in wider population studies. We show that the uncloned profile commonly represents the most common MLG in the infrapopulation with lesser, proportionate additions from less frequent genotypes. Finally we evaluate the implications of within-host diversity in TcI in terms of our understanding of parasite transmission, population genetic structure and the selective forces acting on the parasite.

## 2 Materials and methods

### 2.1 Isolation of *T. cruzi* clones

A total of 211 biological clones were derived from eight isolates (Table 1) using a solid phase medium cloning technique as described in Yeo et al. (2007). Log-phase uncloned culture density was measured using a haemocytometer, and 10^3^–10^4^ cells inoculated from cultures that showed little or no clumping. Isolates were selected on the basis of multilclonality inferred from a multiple (⩾3) alleles at individual loci in their uncloned state (Llewellyn et al., 2009b). Four isolates were selected from the population showing most multiple alleles (*VEN*_Silv_) and two from populations showing fewer (*BOL*_North_ and *BRAZ*_North-East_) (Llewellyn et al., 2009b). Among these eight, two isolates were selected that showed no multiple alleles (XE5167 and M16) to evaluate the potential for ‘hidden multiclonality’ in apparently diploid uncloned profiles.

### 2.2 PCR amplification of microsatellite loci

Nine previously described microsatellite loci were amplified across all clones (Llewellyn et al., 2009b (Supplementary Table S1)). Markers were selected on the basis of the frequency of multi-allelic samples at that locus present in the original study. The following reaction cycle was implemented: a denaturation step of 4 min at 95 °C, 30 amplification cycles (95 °C for 20 s, 57 °C for 20 s, 72 °C for 20 s) and a final 20 min elongation step at 72 °C. With a final volume of 10 ul, 1× ThermoPol Reaction Buffer (New England Biolabs (NEB), UK), 4 mM MgCl_2_, 34 uM dNTPs; 0.75 pmols of each primer, 1 unit of *Taq* polymerase (NEB, UK) and 1 ng of genomic DNA were added. Five fluorescent dyes were used to label forward primers – 6-FAM and TET (Proligo, Germany), NED, PET and VIC (Applied Biosystems, UK). Allele sizes were determined using an automated capillary sequencer (AB3730, Applied Biosystems, UK), manually checked for errors and typed “blind” to control for user bias.

### 2.3 Analysis of multilocus microsatellite profiles

Pair-wise genetic distance between all clone corrected MLGs within each infrapopulation was estimated using *D*_AS_ as described previously (Llewellyn et al., 2009b) and calculated in MICROSAT (Minch et al., 1995). Multi-allelic loci among aneuploid clones were also accommodated as in Llewellyn et al., 2009b. Individual-level genetic distances were calculated as the mean across 1000 re-sampled datasets and the overall *D*_AS_ calculated as the arithmetic mean over all pair-wise distances in the infrapopulation. Among clone corrected diploid MLGs a number of parameters were estimated. *F*_IS_, a measure of the distribution of heterozygosity within and between individuals, was estimated per locus in each group of unique MLGs from each population in FSTAT 2.9.3.2 (Goudet, 1995), as well as over populations over loci using Weir and Cockerman’s estimator *f* in the same package. Among Venezuelan MLGs, subdivision between clone corrected infrapopulations was assessed in Arelquin 3.1 (Excoffier et al., 2005) using an Analysis of Molecular Variance (AMOVA) to determine whether a significant proportion of variance could be attributed to grouping MLGs by host origin. Finally, the extent of multilocus linkage disequilibrium was assessed using the Index of Association (*I*_A_) in each population.

## 3 Results

### 3.1 Infrapopulation diversity

Forty-nine unique MLGs were identified among 211 clones analysed (Table 1). A broadly proportional relationship existed between the number of multi-allelic loci in the non-cloned profile (Original MAL) and the number of distinct MLGs in the sample of the resultant infrapopulation (G), and all infrapopulations contained at least two distinct clones. Importantly, however, multi-allelic profiles in the uncloned isolate were not a prerequisite to observing diversity in the infrapopulation. Among XE5167 clones, for example, 10 distinct MLGs were observed, whilst no multi-allelic loci were observed in the uncloned sample. Additionally, neither time since isolation nor method of isolation seemed to have an observable effect on the number of distinct MLGs present in the sample. Considerable mean genetic divergence (*D*_AS_ > 0.5) between unique MLGs was observed in some isolates (Table 1) and broadly corresponded to the number of distinct MLGs present.

### 3.2 Infrapopulation clonal composition

**Fig. 1:**
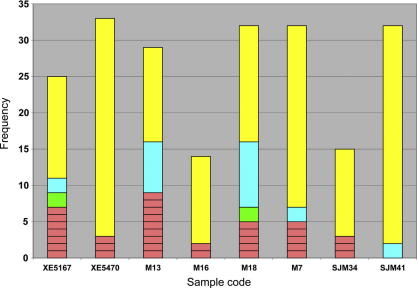
Clonal composition of different intra-host *Trypanosoma cruzi* infrapopulation samples. Composite bars indicate frequency of different multilocus genotypes (MLGs) per isolate. Each division represents a different MLG frequency class. Yellow (light grey) bars indicate dominant/majority MLGs (*n* = 2–30), blue and green (dark grey and black) bars indicate MLGs at lower frequency (*n* = 2–9), and pink bars (medium grey) indicate MLGs represented by a single clone. (For interpretation of the references to colour in this figure legend, the reader is referred to the web version of this article.)

**Fig. 2:**
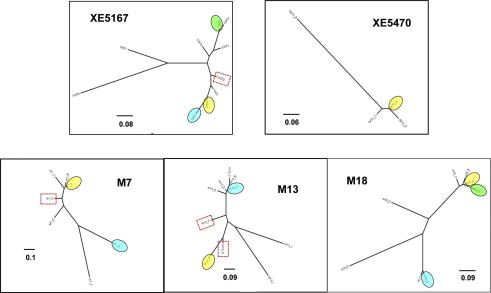
Un-rooted Neighbour-Joining (NJ) trees based on pair-wise *D*_AS_ values between multilocus genotypes MLGs from five parasite infrapopulations where >4 MLGs were identified. *D*_AS_ values were calculated as in Llewellyn et al., 2009b in MICROSAT and PHYLIP v3.6 (272, 280). Sample codes correspond to those in Table 1. Coloured ellipses represent genotypes at a frequency >1 and correspond to Fig. 1. Dashed boxes indicate multilocus aneuploid clones.

Fig. 1 demonstrates the relative abundance of distinct MLGs within each infrapopulation sample. Among each group of MLGs a majority genotype was easily identifiable. Abundant secondary genotypes occurred only in M13 and M18. However, evenly balanced MLG frequencies were not observed within any infrapopulation samples. With the aim of evaluating how multiclonality affects the composite genotype derived from the primary culture, an estimate of the relative contribution of each MLG in each infrapopulation to the original, uncloned microsatellite profile was derived. This value was defined as the proportion of alleles shared (*P*_S_) between each MLG and the composite genotype, as a function of those not shared (*P*_S_ × (1 − *P*_NS_)). A significant linear relationship existed between this estimate and the relative frequency of the corresponding MLG in the infrapopulation sample (*R*_XY_ = 0.367, *P* < 0.012), indicating that the composite genotype was generally most similar to the most abundant MLG in the infrapopulation. Neighbour-Joining (NJ) trees drawn from pair-wise *D*_AS_ values (Fig. 2) also revealed that the most frequent MLGs (Supplementary Table S2) within an infrapopulation were generally highly divergent and that infrapopulations commonly represented a mixture of both closely and distantly related MLGs (Fig. 2).

### 3.3 Aneuploidy, fusion and linkage

Multiple aneuploid clones were isolated across those infrapopulations studied (Table 1). Among these were a number of putative hybrids with identifiable parental types in the same infrapopulation (Supplementary Fig. S1). These were examined under the proposed ‘fusion-then-loss’ model for genetic exchange in *T. cruzi* (Gaunt et al., 2003). However, we could not rule out artefactual hybrid-like profiles associated with plate cloning. In practice this meant that microsatellite peak intensity deviation from hybrid copy number expectations led to the exclusion of all ‘hybrids’ as potential cloning artefacts i.e. mixes of two divergent clones (Supplementary Fig. S1). This phenomenon also meant that MLG diversity in infrapopulations containing ‘aneuploid’ clones may have been marginally over-estimated. In Fig. 2 these isolates cluster between the two most common diploid MLGs, as would be expected if they represent a mixture or hybrid between two distinct clones. Importantly, strong linkage disequilibrium among diploid MLGs (Supplementary Table S2) within each population, as well as, crucially, multiple identical genotypes, supported widespread clonality, not frequent recombination (*VEN*_Silv_ *I*_A_ = 2.54, *P* < 0.001; *BOL*_North_ *I*_A_ = 6.07, *P* < 0.001; *BRAZ*_North-East_ *I*_A_ = 3.14, *P* < 0.001).

### 3.4 Heterozygosity and infrapopulation subdivision

Groups of clone corrected diploid MLGs from each population all demonstrated excess homozygosity, as indicated by positive *F*_IS_ values over loci (Mean *F*_IS_ ± SE: *VEN*_Silv_ = 0.111 ± 0.07; *BOL*_North_ = 0.325 ± 0.14; *BRAZ*_North-East_ = 0.335 ± 0.15) as well as by bootstrapping over populations (Weir and Cockerman’s unbiased estimator *f* = 0.191 (95% Confidence Interval (95% C.I.) 0.038–0.333)). An AMOVA across infrapopulations from *VEN*_Silv_ did demonstrate significant structuring by individual host (*F*_ST_ = 0.175, *P* < 0.0001, 1023 random permutations), and correspondingly a Wahlund effect may be depressing levels of heterozygosity in this population.

## 4 Discussion

We believe this study demonstrates for the first time the extraordinary wealth of parasite genetic diversity that can exist within a single wild reservoir host of *T. cruzi* I. Surprisingly, on the basis of the current dataset, the method of parasite isolation as well as the time since isolation has had little bearing on the number of different MLGs present in each sample. Population statistics, including linkage and heterozygosity indices, when calculated across clones, approximate those observed in the same populations of uncloned isolates (Llewellyn et al., 2009b). The presence of a majority genotype in each sample, and the correlation between the abundance of a genotype in an infrapopulation and its contribution to the uncloned microsatellite profile, to some extent validate the conclusions drawn from population genetic studies that involve uncloned isolates or ‘clonal composites’.

It is reasonable to assume that the parasite diversity represented here comprises barely a fraction of the total potentially present within each individual host. Presumably, continual cloning and genotyping would uncover novel genotypes at a rate of approximately one in every four (49/211 – Table 1) clones analysed. Serial sampling of individual hosts at different time intervals and from different tissues (e.g. Valadares et al., 2008) would likely reveal further genotypes. Solid phase cloning may be too imprecise to evaluate such diversity. Whilst ‘fusion-recombinant’ artefacts can be identified and excluded, other phenomena, especially differential in vitro growth rates between clones, introduce unquantifiable sample error. There are a number of promising new technologies to address these issues, particularly Whole Genome Amplification, which can now be effectively targeted at individual cells (e.g. Kwon and Cox, 2004). However, the problem of isolating individual parasite cells directly from blood and tissue remains.

Culture bias is a ubiquitous issue in all microbial sampling. In *T. cruzi* such bias is aggravated in mammalian samples by low circulating parasitemia. From the correlation between MLG abundance and contribution to the uncloned microsatellite profile, we can at least infer the same in vitro growth bias between MLGs in strains grown in liquid culture as those grown on solid phase medium. Thus each uncloned genotype represents most closely the majority genotype in the infrapopulation, not a balanced mix between MLGs, as in models proposed for *Trypanosoma congolense* (Holzmuller et al., 2010). In population genetic terms, this implies that a ‘population’ comprising multiple uncloned isolates approximates a population of clones, each clone taken from a different host. This also supports pioneering work by Finley et al. (1987) who modeled growth of *T. cruzi* clonal mixtures in culture and found that a single clone predominated, although Finley did not predict that divergent strains could also persist at lower frequencies (Finley et al., 1987).

Using simulated samples containing 10 infrapopulations from which 20 clones each were drawn, Prugnolle and De Meeus (2010) suggest that, by comparison, the inclusion of only a single clone per host or vector in a population study (modeled as 20 clones, one each from 20 infrapopulations) can artefactually lower observed heterozygosity (increase *F*_IS_) and reduce multilocus linkage (*r*_D_ which approximates *I*_A_) compared with treating each infrapopulation separately. Our sample sizes are too low to test this assertion empirically, largely because the high frequency of repeated genotypes vastly inflates the sample effort required to generate more than a single unique MLG per sample, an illustration of how difficult it is to test model-derived assertions in the field properly. However, when we grouped multiple clones from different hosts from the same study sites together, both linkage and heterozygosity statistics mirrored those which we observed previously (Llewellyn et al., 2009b).

Reconciling the maintenance of high infrapopulation diversity with what is understood about the transmission dynamics, genetics and biology of *T. cruzi* is a complex task. De novo mutation within the host is an unlikely explanation. The presence of highly divergent genotypes within the same host suggests frequent introduction of diversity into each infrapopulation via migration (i.e. transmission), not mutation. Interestingly, however, we cannot rule out that groups of closely related MLGs also observed within each infrapopulation (Fig. 2) may share an ancestor within the same host, or perhaps the same host pedigree, if congenital infection represents an important means of transmission between sylvatic reservoirs, as is the case in some human populations (e.g. Bern et al., 2009).

Genetic recombination, now known to occur in *T. cruzi* in vitro (Gaunt et al., 2003), as well as at both species (Machado and Ayala, 2001) and population (Ocaña-Mayorga et al., 2010) levels, does not seem a relevant source of multiclonality in the context of these data. Linkage disequilibrium among diploid MLGs from each population, as well as multiple instances of identical MLGs, suggest a predominantly clonal mode of reproduction. Furthermore, the putative fusion recombinants indentified could not be confidently distinguished from cloning artefacts.

Criscione and Blouin (2006) suggest that clonal diversity can be maintained at the infrapopulation level given high rates of parasite transmission and correspondingly minimal MLG extinction. Poisson-distributed allele frequencies in *VEN*_Silv_, *BOL*_North_ and *BRAZ*_North_ are also consistent with low rates of clonal extinction (Llewellyn et al., 2009b). Subdivision by host among infrapopulations from *VEN*_Silv_, while statistically significant in this study and consistent with low transmission, receives little support from previous work on *T. cruzi* at the intra-DTU level (Spotorno et al., 2008; Llewellyn et al., 2009b; Miles et al., 2009), where diversification by host is not observed. Instead this result is likely to reflect error associated with small infrapopulation sample sizes. Thus the balance of evidence still favours high sylvatic transmission rates in *T. cruzi*. Paradoxically, the stercorarian route via which *T. cruzi* infects its hosts is highly inefficient (estimated at one infectious contact in 650 triatomine feeds (Rabinovich et al., 1990)). However, the associated signature of serial population reductions and clonal extinctions is absent from our data. High clonal and genetic diversity are instead consistent with en masse transmission of parasite populations from vector to host. Oral transmission, increasingly reported in human populations (Pereira et al., 2009), could play a role, perhaps during opportunistic predation of triatomines. Recent experimental work in *T. brucei* shows that bottlenecks do occur during transmission within the tsetse vector (Oberle et al., 2010). Similar experimental study of the impact of stercorarian transmission on *T. cruzi* clonal diversity is required, including a comparison with the oral route.

To maintain the level of diversity and multiclonality observed in sylvatic TcI, oral transmission would need to occur at considerable frequency in both space and time. Instead, interaction between parasite and host could play a role. Microsatellite diversity in a clonal organism cannot be considered strictly neutral, even it occurs outside protein coding regions. This derives from the observation that microsatellite loci can appear non-neutral through genetic linkage, the so-called ‘hitch-hiking’ effect. Thus the entire *T. cruzi* genome is the selective unit in clonal populations. It is, therefore, conceivable that high infrapopulation diversity observed in this study at microsatellite loci may be indirectly maintained through selection. Such diversification reflects the heterogeneity of the host environment and the numerous cell types which *T. cruzi* infects, and there is limited evidence that different DTUs can sequester in different tissue types within the same host (e.g. Burgos et al., 2008; Valadares et al., 2008). Host immune pressure could also contribute, although *T. cruzi* may not experience such intense exposure as extracellular trypanosomes such as *Trypanosoma brucei*, where the mechanism for immune avoidance is more clearly defined.

Resolving the interplay between selection and transmission, as well the underlying genetic mechanism accounting for the coexistence of so many unique clones within individual *T. cruzi* reservoir hosts is beyond the scope of this study. Other work suggests that mismatch repair inefficiency may play an important role in generating antigenic variation in *T. cruzi* (Machado et al., 2006). If so, hypervariability at microsatellite loci could represent molecular fallout from such an adaptive strategy. Fortunately, many of the tools are now in place to enable carefully designed field and experimental work to test such assertions. This study provides a qualitative insight into the implications multiclonality has for population genetic studies as well as the first glimpse at the extraordinary levels of diversity that exist among *T. cruzi* clones within their ancient reservoir hosts.

## Figures and Tables

**Supplementary Figure 2:**
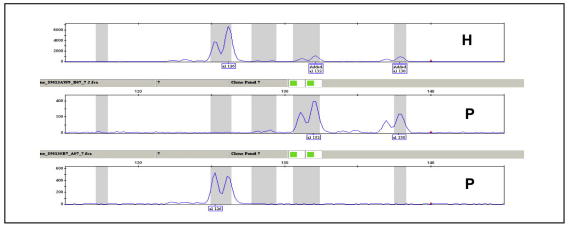
An example of a putative *Trypanosoma cruzi* cloning artefact that resembles 409 parental (P) and hybrid (H) allele combinations under a fusion-then-loss model of genetic exchange. Peak 410 intensities, however, are not consistent with the expected copy number ratio for either tetrasomic (2:1:1) 411 or trisomic (1:1:1) progeny at this locus.

**Table 1:** Samples cloned in this study, multiallellic loci and multiclonality.

Sample code	Host^a^	Date	Locality^b^	Isolation method^c^	Original MAL	*n*	G	Mean *D*_AS_ ± S.E.	Clone MAL
XE5167	Dm	14.09.99	Pa/Br	XE	0	25	10	0.399 ± 0.035	4
XE5740	Dm	18.6.02	Pa/Br	XE	2	33	4	0.295 ± 0.10	0
M13	Dm	12.6.04	Ba/Ve	B	7	29	10	0.509 ± 0.042	5^d^
M16	Dm	13.6.04	Ba/Ve	B	0	14	3	0.067 ± 0.017	0
M18	Dm	13.6.04	Ba/Ve	XE	5	32	8	0.523 ± 0.053	1
M7	Dm	14.5.04	Ba/Ve	B	4	32	7	0.591 ± 0.074	2^d^
SJM34	Dm	7.9.04	Be/Bo	XE	4	14	3	0.583 ± 0.267	1
SJM41	Po	9.9.04	Be/Bo	B	2	32	2	0.056	0
					Total:	211	49		

a Dm – *Didelphis marsupialis*; Po – *Philander opossum.*

b Pa/Br – Para, Brazil (*VEN*_Silv_); Ba/Ve – Barinas, Venezuela (*BRAZ*_North-East_); Be/Bo – Beni, Bolivia (*BOL*_North_). Population names correspond to those in Llewellyn et al. (2009b).

c XE – Xenodiagnosis, B – Blood agar culture. Original MAL – Number of multi-allelic (⩾3) loci present in uncloned sample. *n* – Number of clones analysed. *G* – Number of distinct multilocus genotypes (MLGs) present. Mean *D*_AS_ – Mean pair-wise genetic distance between distinct MLGs from each host ± S.E. about the mean. Includes aneuploid MLGs calculated as a mean over 1000 diploid resamplings (see Materials and methods). Clone MAL – number of clones containing multi-allelic loci.

d Samples that include identical aneuploid genotypes represented more than once in the dataset.
