# Supplementary material for: Extraordinary Trypanosoma cruzi diversity within single mammalian reservoir hosts implies a mechanism of diversifying selection
Source: Int J Parasitol. 2011 May;41(6-10):609–14. doi: 10.1016/j.ijpara.2010.12.004 (PMC3084450; doi:10.1016/j.ijpara.2010.12.004)
Supplement: Supplementary data 1 — Supplementary tables [file mmc1.doc]

**Supplementary Table S1.** Microsatellite loci analysed in this study.

| Primer  Code^a^ | Chromosome^b^ | Repeat | Forward Primer/Reverse primer |
| --- | --- | --- | --- |
| 7093(TA)c^c^ | TcChr39-S | (TA)n | CCAGTTTCACACATACGCAA |
|  |  |  | CGTTTGGAGGAGGATTGAGA |
| 10359(TTG) | TcChr41-S | (TTG)n | ATGGGTGCGAGAGGTATGTC |
|  |  |  | TGTCAAAACAGCGGAAAGAA |
| 6855(TA)(GA) | TcChr10-S | (TA)n(GA)n | TGTGATCAACGCGCATAAAT |
|  |  |  | TTCCATTGCCTCGTTTTAGA |
| 10187(GA) | TcChr37-P | (GA)n | GTCACACCACTAGCGATGACA |
|  |  |  | ACTGCACAATACCCCCTTTG |
| 10187(TTA) | TcChr37-P | (TTA)n | GAGAGAGATTCGGAAACTAATAGC |
|  |  |  | CATGTCCCTTCCTCCGTAAA |
| 6925(TG)a | TcChr39-S | (TG)n | TCGTTCTCTTTACGCTTGCA |
|  |  |  | TAGCAGCACCAAACAAAACG |
| 11283(TCG) | TcChr40-P | (TCG)n | ACCACCAGGAGGACATGAAG |
|  |  |  | TGTACACGGAACAGCGAAG |
| 11283(TA)b | TcChr40-P | (TA)n | AACATCCTCCACCTCACAGG |
|  |  |  | TTTGAATGCGAGGTGGTACA |
| 8741(CT)(TA) | TcChr24-P | (CT)n(TA)n | GCAGAGACGCACAGACACAT |
|  |  |  | AAAGTGCCATCCCACCCTC |

^a^ Llewellyn et al., 2009b

^b^ Weatherly et al., 2009

^c^Flanking region longer that 7093(TA)c as published in Llewellyn et al., 2009b

**References**

Llewellyn, M.S., Miles, M.A., Carrasco, H.J., Lewis, M.D., Yeo, M., Vargas, J., Torrico, F., Diosque, P., Valente, V., Valente, S.A., Gaunt, M.W., 2009b. Genome-scale multilocus microsatellite typing of *Trypanosoma cruzi* discrete typing unit I reveals phylogeographic structure and specific genotypes linked to human infection. PLoS Pathog 5, e1000410.

Weatherly, D.B., Boehlke, C., Tarleton, R.L., 2009. Chromosome level assembly of the hybrid *Trypanosoma cruzi* genome. BMC Genomics 10, 255.

**Supplementary Table S2.** Microsatellite allele sizes for diploid multilocus genotypes (MLGs) analysed in this study. Aneuploid clones are excluded.

|  | **Locus** | | | | | | | | |
| --- | --- | --- | --- | --- | --- | --- | --- | --- | --- |
|  | 6855(TA)(GA) | 6925(TG)a | 7093(TA)c | 8741(CT)(TA) | 10187(GA) | 10187(TTA) | 10359(TTG) | 11283(TCG) | 11283(TA)b |
| **MLG** |  |  |  |  |  |  |  |  |  |
| m13a | 153/157 | 138/142 | 105/115 | 154/156 | 110/116 | 168/178 | 97/108 | 129/132 | 175/175 |
| m13g | 153/157 | 138/142 | 105/115 | 156/156 | 110/116 | 168/168 | 97/108 | 129/132 | 175/175 |
| m13h | 153/155 | 140/140 | 105/110 | 145/145 | 103/109 | 173/173 | 112/112 | 126/126 | 173/173 |
| m13i | 153/157 | 138/142 | 105/115 | 156/156 | 110/116 | 168/178 | 97/108 | 129/132 | 175/175 |
| m13j | 153/157 | 138/142 | 105/115 | 154/152 | 110/116 | 168/178 | 97/108 | ND | 175/175 |
| m16a | 155/157 | 136/138 | 107/109 | 152/152 | 113/116 | 170/170 | 112/112 | 129/129 | 171/177 |
| m16b | 155/157 | 136/138 | 107/109 | 152/156 | 113/116 | 170/170 | 112/112 | 129/129 | 171/177 |
| m16c | 155/157 | 138/138 | 107/109 | 152/152 | 113/116 | 170/170 | 112/112 | 129/129 | 171/177 |
| m18a | 149/155 | 138/138 | 105/115 | 147/147 | 113/116 | 175/179 | 108/112 | 129/129 | 171/177 |
| m18c | 149/155 | 138/138 | 105/115 | 147/147 | 113/116 | 173/179 | 108/112 | 129/129 | 171/177 |
| m18e | 147/153 | 140/140 | 105/105 | 145/150 | 103/109 | 173/173 | 110/110 | 132/138 | 175/175 |
| m18f | 149/155 | 138/138 | 105/115 | 147/147 | 113/116 | 175/175 | 108/112 | 129/129 | 171/177 |
| m18g | 153/157 | 138/142 | 105/115 | 154/156 | 110/116 | 168/178 | 97/106 | 129/132 | 175/175 |
| m18h | 153/157 | 138/142 | 105/115 | 154/156 | 110/116 | 168/178 | 97/108 | 129/132 | 175/175 |
| m18i | 149/151 | 136/138 | 105/107 | 143/147 | 110/113 | ND | 106/112 | 118/129 | 175/175 |
| m7a | 149/149 | 136/140 | 107/109 | 154/156 | 113/113 | 168/175 | 108/112 | 132/132 | 171/175 |
| m7c | ND | ND | 107/109 | 154/156 | 113/113 | 168/173 | 108/112 | ND | 175/175 |
| m7e | 149/149 | 138/140 | 105/107 | 147/154 | 113/116 | 170/175 | 108/112 | 132/132 | 175/177 |
| m7f | 149/149 | 136/140 | 107/109 | 154/156 | 113/113 | 175/178 | 108/112 | 132/132 | 171/175 |
| m7g | 153/155 | ND | 105/110 | 145/145 | 103/109 | 173/173 | 110/110 | 126/126 | 173/173 |
| sjm34a | 136/136 | 140/142 | 113/115 | 150/154 | 109/109 | 168/170 | 106/110 | 129/129 | 171/177 |
| sjm34c | 149/149 | 136/140 | 107/109 | 154/156 | 113/113 | 168/175 | 108/112 | 132/132 | 171/175 |
| sjm34d | 136/136 | 140/142 | 113/115 | 150/154 | 109/109 | 168/170 | 106/110 | 129/129 | 171/171 |
| sjm41a | 153/155 | 131/131 | 107/110 | 145/152 | 107/107 | 173/173 | 106/106 | 126/126 | 175/183 |
| sjm41b | 153/155 | 131/131 | 107/107 | 145/152 | 107/107 | 173/173 | 106/106 | 126/126 | 175/183 |
| 5167c | 136/136 | 140/140 | 105/107 | 143/143 | 103/105 | 166/173 | 110/110 | 132/132 | 173/173 |
| 5167d | 153/155 | 140/140 | 105/110 | 145/145 | 103/109 | 173/173 | 110/110 | 126/126 | 173/173 |
| 5167e | 147/153 | 140/140 | 105/105 | 145/150 | 103/109 | 173/166 | 110/110 | 132/138 | 175/175 |
| 5167f | 153/155 | 140/140 | 105/110 | 145/145 | 103/109 | 173/173 | 112/112 | 126/126 | 173/173 |
| 5167g | 153/157 | 138/142 | 105/115 | 156/156 | 110/116 | 168/178 | 97/108 | 129/132 | 175/175 |
| 5167i | 149/149 | 138/140 | 105/107 | 143/143 | 113/115 | 168/178 | 112/112 | 126/129 | 175/179 |
| 5470a | 136/136 | 140/140 | 105/107 | 143/143 | 103/105 | 166/173 | 110/110 | 132/132 | 173/173 |
| 5470b | 136/136 | 140/140 | 105/107 | 143/143 | 103/105 | 166/166 | 110/110 | 132/132 | 173/173 |
| 5470c | ND | 140/140 | 105/107 | 143/143 | 103/105 | 166/173 | 110/110 | 132/132 | 173/173 |
| 5470d | 153/155 | 140/140 | 105/110 | 145/145 | 103/109 | 173/173 | 110/110 | 126/126 | 173/173 |

ND, no data.
